# Monitoring for glaucoma progression with SAP, electroretinography (PERG and PhNR) and OCT

**DOI:** 10.1007/s10633-021-09854-8

**Published:** 2021-10-15

**Authors:** Barbara Cvenkel, Maja Sustar, Darko Perovšek

**Affiliations:** 1 Department of Ophthalmology, University Medical Centre Ljubljana, Grabloviceva 46, 1000 Ljubljana, Slovenia; 2 Faculty of Medicine, University of Ljubljana, Vrazov trg 2, 1000 Ljubljana, Slovenia

**Keywords:** Glaucoma, Monitoring, Optical coherence tomography, Pattern electroretinography, Photopic negative response

## Abstract

**Purpose:**

To investigate the value of pattern electroretinography (PERG) and photopic negative response (PhNR) in monitoring glaucoma compared to standard clinical tests (standard automated perimetry (SAP) and clinical optic disc assessment) and structural measurements using spectral-domain OCT.

**Methods:**

A prospective study included 32 subjects (32 eyes) with ocular hypertension, suspect or early glaucoma monitored for progression with clinical examination, SAP, PERG, PhNR and OCT for at least 4 years. Progression was defined clinically by the documented change of the optic disc and/or significant visual field progression (EyeSuite™ trend analysis). One eye per patient was included in the analysis.

**Results:**

During the follow-up, 13 eyes (40.6%) showed progression, whereas 19 remained stable. In the progressing group, all parameters showed significant worsening over time, except for the PhNR, whereas in the stable group only the OCT parameters showed a significant decrease at the last visit. The trend of change over time using linear regression was steepest for the OCT parameters. At baseline, only the ganglion cell complex (GCC) and peripapillary retinal nerve fibre (pRNFL) thicknesses significantly discriminated between the stable and progressing eyes with the area under the ROC curve of 0.72 and 0.71, respectively. The inter-session variability for the first two visits in the stable group was lower for OCT (% limits of agreement within ± 17.4% of the mean for pRNFL and ± 3.6% for the GCC thicknesses) than for ERG measures (within ± 35.9% of the mean for PERG N95 and ± 59.9% for PhNR). The coefficient of variation for repeated measurements in the stable group was 11.9% for PERG N95 and 23.6% for the PhNR, while it was considerably lower for all OCT measures (5.6% for pRNFL and 1.7% for GCC thicknesses).

**Conclusions:**

Although PERG and PhNR are sensitive for early detection of glaucomatous damage, they have limited usefulness in monitoring glaucoma progression in clinical practice, mainly due to high inter-session variability. On the contrary, OCT measures show low inter-session variability and might have a predicting value for early discrimination of progressing cases.

**Supplementary Information:**

The online version contains supplementary material available at 10.1007/s10633-021-09854-8.

## Introduction

Monitoring patients with glaucoma to detect progression and determine the rate of visual function loss is the mainstay of glaucoma care. In clinical settings, the recommended tests for monitoring include tonometry, clinical examination of the optic disc and retinal nerve fibre layer (RNFL), and visual field testing. The evaluation of structural changes is complemented by quantitative measurement using optical coherence tomography (OCT). Standard automated perimetry (SAP) is the reference standard for assessment of visual function in glaucoma [1]. It is a subjective method relying on patients’ cooperation, and in some patients there is a high variability of mean deviation over time which decreases the ability to detect true change from noise [2]. Electroretinography (ERG) is an objective method, and both pattern ERG (PERG) and the photopic negative response (PhNR) of the ERG are sensitive markers of the retinal ganglion cell (RGC) dysfunction that is a characteristic of glaucoma [3–6].

The PERG is a measure of the electrical activity of the RGC population of the central retina (more than 40% of the total RGC population) in response to a suprathreshold stimulus [7]. The PhNR of the light-adapted ERG is a negative-going wave that occurs after the b-wave in response to a brief flash. It reflects generalized activity of the RGC and their axons [8], and its amplitude, similarly to PERG, can be reduced early in diseases that affect the innermost retina [5].

A recent review article on the clinical applicability of electrophysiological tests in glaucoma found a reasonable correlation between amplitudes and latency of electrophysiological measures and routine tests for glaucoma, mainly SAP and OCT [9]. However, it remains unclear what is the role of these tests in early detection and monitoring of glaucoma. Requirement of complex protocols, equipment and experienced personnel limits the use of electrophysiology to special cases and research. In our previous cross-sectional study, we found that patients with suspect and early glaucoma had significantly reduced PERG N95 and PhNR amplitudes compared to controls, indicating high sensitivity of both electrophysiological measures for early detection of ganglion cell damage [10]. In addition, in eyes with suspect glaucoma, a greater decrease in PhNR amplitude was associated with small changes in peripapillary retinal nerve fibre layer (pRNFL) thickness that may be predictive of glaucoma progression [10].

The aim of the present study was to investigate the value of PERG and PhNR in monitoring glaucoma compared to standard clinical tests (SAP and optic disc assessment) and structural measurements using spectral-domain OCT.

## Methods

This longitudinal study was performed according to the tenets of the Declaration of Helsinki and was approved by the National Ethics Committee, University Medical Centre Ljubljana, Ljubljana, Slovenia (KME 33/11/11). All of the participants were fully informed about possible consequences of the research protocol and signed their informed consent before enrolment.

Thirty-two patients with ocular hypertension (OHT), suspect glaucoma or early open-angle glaucoma were recruited from the Glaucoma Clinic of the Department of Ophthalmology, University Medical Centre Ljubljana, Slovenia. The enrolment started in January 2012, and the participants had at least 4 years of follow-up. The patients were aged 25 to 81 years (mean age ± SD, 59.5 ± 12.0 years), with 9 males and 23 females. The inclusion criteria were visual acuity ≥ 0.8 Snellen, clear optic media and myopia < −5D. Exclusion criteria were treatment with topical or systemic corticosteroids, diabetes or neurological disorders (e.g. Parkinson’s disease, multiple sclerosis).

At baseline, all of these patients underwent complete ophthalmological examination and visual field testing and were diagnosed as: OHT, characterized by untreated intraocular pressure (IOP) consistently > 21 mmHg, and normal optic disc and visual field; suspect glaucoma, characterized by suspicious appearing optic disc, with normal or suspicious visual field; or early glaucoma, characterized by the presence of glaucomatous changes at the optic disc, and corresponding reproducible visual field loss, with mean defect from 2 to 6 dB. A glaucomatous optic disc appearance included focal and/or diffuse thinning of the neuroretinal rim, and asymmetry in the optic disc cupping between the eyes > 0.2 that was not caused by the difference in optic disc size or shape [11]. Due to the large variation of optic disc appearance among healthy subjects (in size, shape), there are no clear criteria for early glaucomatous disc changes. Therefore, the term suspicious optic disc was used when the discs had features resembling glaucomatous optic disc changes and a definite diagnosis of glaucoma can only be ascertained with the follow-up [11]. The assessment of optic disc was performed by a glaucoma consultant (BC) using the above criteria.

Visual field defects were defined as three or more adjacent points of ≥ 5 dB loss or two or more points ≥ 10 dB loss, in the absence of other changes that could explain the defect.

The patients with glaucoma and high-risk OHT were treated with topical hypotensive medication, either as a monotherapy or as a combination of drugs, such as prostaglandin analogues, beta-blockers, alpha-2 agonists, and carbonic anhydrase inhibitors.

After ophthalmological examination, IOP measurement and visual field testing, all subjects underwent the ERG and OCT tests.

### Visual field testing

Standard automated perimetry (SAP) was performed in all subjects, using an Octopus 900 perimeter (Haag-Streit AG, Koeniz, Switzerland) with the Dynamic Strategy G2 program. Only reproducible tests with < 20% false-positive and < 20% false-negative response rates were used in the evaluation. The following global visual field indices were recorded: mean defect (MD), and square root of loss variance (sLV). The MD is a positive value using Octopus perimetry and represents the average visual field loss from all locations, whereas sLV is a measure of variability across the visual field and increases in localized defects.

### Electroretinography

Electroretinographic responses were recorded using an Espion visual electrophysiology testing system (Diagnosys LLC, Littleton, MA, USA). The recording procedure for the PERG and the PhNR followed the standards and guidelines of International Society for Clinical Electrophysiology of Vision (ISCEV) [12, 13]. A HK loop served as a recording electrode and was placed in the fornix of the lower eyelid [14]. The silver chloride reference electrode was placed on the ipsilateral temple, and the ground electrode was positioned on the forehead. The PERG recording does not require pupil dilation; therefore, it was recorded first. It was elicited with a 0.8° checkerboard pattern with 99% contrast that reversed 1.8 times per second, which was presented on a 21.6° X 27.8° cathode ray tube screen stimulator. The patients were sitting 1 m away from the screen stimulator and used optimal refractive correction during the recording. One hundred sweeps were collected for each recording and repeated at least twice. Later, the pupils were dilated with 1% tropicamide (Mydriacyl, Alcon) and the patients were light-adapted for 10 min. Photopic ERGs were elicited with a Ganzfeld ColorDome stimulator (Diagnosys LLC, Littleton, MA, USA), using 2.5 cd s/m2 monochromatic red stimuli (635 nm) on a 10 cd/m2 blue background (470 nm). The rate of stimulation was 1 Hz, and 30 sweeps were collected for each recording, which was repeated at least three times. Sweeps that included artefacts with an amplitude larger than 500 μV were rejected automatically during the recording, while sweeps with low-amplitude artefacts that influenced the baseline or the expected waveform of the response up till 80 ms after the stimulus onset were rejected manually. The average of two most repeatable or all three recordings (collected from 50–80 sweeps) was taken into further analysis. The signals were amplified with a band pass from 0.1 to 500 Hz. For the PERG, the P50 amplitude was measured from the N35 trough, while the N95 amplitude was measured from the P50 peak. For the photopic ERG, the PhNR amplitude was measured from the baseline to the negative trough that clearly appeared after the b-wave and the i-wave. The ratio between the PhNR and b-wave amplitude (PhNR ratio = PhNR amplitude/b-wave amplitude) was also calculated and used for further analysis.

### OCT measurements

Spectral-domain OCT (Topcon 3D OCT-2000; Topcon Inc., Tokyo, Japan) was performed following the ERG test. The following two scan protocols were used: 6.0 × 6.0 mm three-dimensional (3D) disc (512 A-scans by 128 B-scans) and 6.0 × 6.0 mm 3D macula (512 A-scans by 128 B-scans). The commercial software derives a peripapillary retinal nerve fibre layer (pRNFL) thickness plot from the segmentation of the 3D disc scan, by centring a circle after the scan is obtained. The data were exported by the software and analysed by the proprietor’s automatic segmentation algorithm. The following layers were collected: the mean pRNFL thickness, the mean thicknesses of the macular nerve fibre layer (NFL), the ganglion cell–inner plexiform layer (mGCIPL) and the ganglion cell complex (GCC). Only scans with the image quality > 70 were accepted.

### Follow-up

The patients were examined over the minimum of 4 years, using OCT and electroretinography yearly (within the interval 11–13 months), and clinical examination was performed according to the European Glaucoma Society guidelines within the period 6–12 months. Criteria for glaucoma progression were based on the SAP and/or documented changes of the optic disc/RNFL at ophthalmoscopy from baseline (change in the neuroretinal rim thinning, disc haemorrhage). To define visual field progression, a trend-based analysis was performed in at least six reproducible visual field tests using the commercially available software (EyeSuite Progression Analysis™). Progression was defined as diffuse (MD) and/or local (sLV) worsening at *P *< 1% [15].

### Statistical analysis

The statistical analyses were carried out using the Statistical Package for Social Sciences (SPSS 2013, version 22; Inc., Chicago, Illinois, USA) and Origin 8.0 (OriginLab Corporation, Northampton, USA). Data of paired eyes are likely to be correlated, so by default only the right eye was included in the analysis. The exception were patients with progression in both eyes, for which the eye with faster rate of progression in the visual field was analysed. In a few patients, the left eye was included because of the better ERG signal. The normality of the distributions for dependent variables was tested using the Shapiro–Wilk test. Most variables showed normally distributed data. Therefore, the means’ comparison was made with the analysis of variance (ANOVA) and the Dunnett’s post hoc test. The trends of changes over time for clinical, electrophysiological and OCT parameters were calculated by applying linear regression line: *y* = *a* + *bx,* to the mean values of each parameter over time. The steepness of slope is indicated by the parameter *b* and the parameter *a* is the intercept, indicating the value of each parameter at the time point 0. The calculated fitted lines were compared between the stable and progressing group using the F test to determine whether the two data sets were significantly different from each other and to detect differences in progression over time. Correlations of ERG and OCT measures with the visual field indices were calculated using the Pearson correlation test. The Bland–Altman analysis was used to assess test–retest variability for the first two visits in the stable group, and 95% confidence intervals were constructed to assess the precision of the limits of agreement (LoA), as described by Bland and Altman [16]. LoAs were also calculated as a percentage of the mean value to allow between-session findings to be compared across techniques [17]. The coefficient of variation was calculated in the stable group as the ratio of the standard deviation to the mean value for each parameter at all five visits mainly to compare the difference in the variability among examination methods (ERG, OCT, visual field). To discriminate between the progressing and stable glaucoma eyes at the first visit, receiver operating characteristic (ROC) curves for all the variables were constructed. All the statistical tests were two-sided, and a *p* value < 0.05 was considered statistically significant.

## Results

**Fig. 1:**
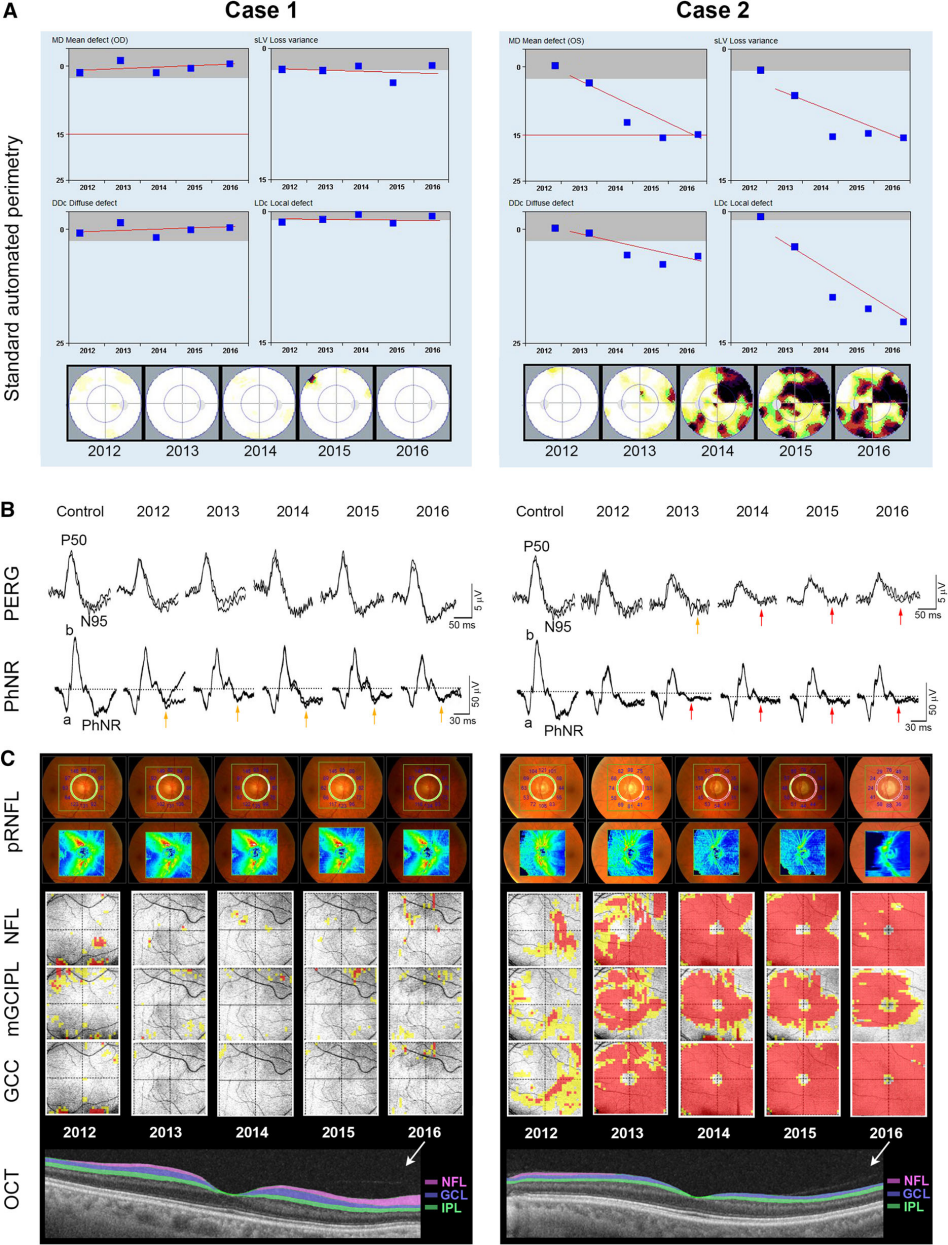
SAP **a**, ERG **b** and OCT **c** findings at follow-up visits for 2 patients, Case 1 with stable clinical picture and Case 2 with fast progression, that was seen in all measures analysed. At the PhNR traces, yellow arrows indicate borderline reduction in the response, while red arrows indicate a notable abnormality of the response

Thirty-two eyes of 32 patients were analysed in this longitudinal study. According to their clinical appearance at baseline, these eyes were classified as OHT (6 eyes), suspect glaucoma (16 eyes) or early glaucoma (10 eyes). The mean follow-up was 52 months (SD 4.7 months; range 48–64 months). Participants’ characteristics and clinical measurements from one eye per subject at baseline are presented in Table 1. During follow-up, 13 patients showed glaucoma progression in both eyes and 19 patients were stable. In Fig. 1, two case examples are shown: case 1 an eye without progression in the visual field, PERG, PhNR and pRNFL thickness, and case 2 an eye with glaucoma progression with early/minimal changes in the visual field (nasal step) and the pRNFL thickness, but at the same time (2013) an important decrease in the PhNR and PERG. With follow-up, significant progression in SAP and OCT was noted, while the ERG abnormality remained stable.

**Table 1:** Clinical characteristics of participants and their measurement data at baseline

No.	Age (yrs)	Gender	Eye	Diagnosis	VA	IOP (mmHg)	MD (dB)	sLV (dB)	N95 (µV)	PhNR (µV)	pRNFL (µm)	mGCIPL (µm)	GCC (µm)	Progression
1	51	F	OD	Suspect	1.0	19	0.4	3.2	4.9	11.9	79	65	99	No
2	66	M	OD	Glaucoma	1.0	17	1.5	2.1	7.0	27.5	98	68	106	No
3	55	F	OD	Suspect	1.0	14	0.5	2.3	6.9	18.2	83	63	102	No
4	75	M	OD	Glaucoma	0.9	18	-0.3	2.5	7.7	14.4	82	62	92	No
5	57	F	OD	OHT	1.0	25	-2.1	1.8	7.0	5.3	89	71	112	No
6	44	M	OD	Suspect	1.0	19	3.0	3.8	6.0	22.8	92	68	115	No
7	57	F	OD	OHT	1.0	19	1.0	2.2	7.0	16.8	96	65	111	No
8	25	F	OD	OHT	1.0	25	0.4	1.9	8.7	31.5	84	69	117	No
9	81	F	OD	OHT	1.0	23	0.6	3.3	6.5	10.9	79	59	100	No
10	54	M	OS	Suspect	1.0	19	2.0	2.8	3.5	7.7	89	61	91	No
11	52	M	OD	OHT	1.0	24	-0.2	2.4	6.5	11.4	81	66	109	No
12	78	F	OD	Suspect	1.0	15	0	2.1	7.0	17.2	91	62	105	No
13	72	M	OS	Suspect	1.0	18	2.7	4.0	5.9	12.9	73	68	100	No
14	43	M	OD	Glaucoma	0.8	13	1.1	2.5	3.6	12.2	67	53	76	No
15	65	F	OD	Suspect	1.0	19	1.4	2.6	4.9	20.6	80	59	95	No
16	61	F	OD	Suspect	1.0	17	0.4	1.8	5.1	11.2	78	58	90	No
17	73	M	OS	Glaucoma	0.9	14	8.5	7.1	4.6	4.5	70	61	87	No
18	65	F	OD	Suspect	1.0	26	3.8	3.8	4.8	5.4	66	59	92	No
19	44	F	OD	OHT	1.0	24	-1.1	2.1	6.9	11.6	86	68	106	No
20	47	F	OD	Suspect	1.0	19	1.1	2.5	4.6	9.7	95	60	95	Yes
21	75	F	OS	Suspect	1.0	18	4.6	4.6	5.0	10.6	74	60	88	Yes
22	66	F	OD	Glaucoma	0.9	25	-0.7	2.0	3.8	10.7	79	57	89	Yes
23	65	F	OD	Glaucoma	0.9	32	2.2	3.7	5.0	7.3	70	76	124	Yes
24	67	F	OS	Glaucoma	0.9	21	4.0	5.2	4.1	15.8	70	64	96	Yes
25	65	F	OD	Glaucoma	0.7	18	3.2	4.0	4.3	9.7	54	60	82	Yes
26	61	F	OS	Suspect	1.0	22	2.0	4.5	6.7	14.9	77	59	90	Yes
27	54	F	OS	Glaucoma	1.0	17	3.8	7.9	5.1	25.8	69	56	80	Yes
28	47	F	OD	Glaucoma	1.0	12	4.7	7.4	5.5	14.7	68	63	86	Yes
29	53	F	OD	Suspect	1.0	25	2.1	2.3	5.9	17.5	76	64	96	Yes
30	64	F	OS	Suspect	0.9	22	0.1	2.3	5.0	21.1	99	65	105	Yes
31	63	F	OS	Suspect	1.0	18	0	2.8	7.1	15.3	76	62	94	Yes
32	59	M	OS	Suspect	1.0	24	-0.7	2.2	6.7	20.9	78	58	90	Yes

F—female, M—male; OD—right eye, OS—left eye; OHT—ocular hypertension, Suspect—suspected glaucoma; pRNFL—peripapillary retinal nerve fibre layer thickness; mGCIPL—macular ganglion cell–inner plexiform layer thickness; GCC—ganglion cell complex thickness

The clinical measurements (visual acuity, IOP, visual field indices), ERG parameters (P50, N95, PhNR, PhNR ratio) and OCT data at baseline, at the third, intermediate visit and at the last follow-up visit for the stable and progressing eyes are summarized in Table 2. For the stable group, all the clinical and electrophysiological parameters, MD, sLV, NFL and GCC thickness remained unchanged during the follow-up, while for the pRNFL and mGCIPL thickness a significant worsening was observed at the last visit only. For the progressing group, a significant worsening was seen for MD, sLV, N95 and all the OCT parameters, while P50 and PhNR showed only slight, but not significant decrease over time of follow-up.

**Table 2:** Means comparison between the first, intermediate (3^rd^) and the last (5^th^) visit for clinical findings, SAP, ERG and OCT measures for the stable (1) and progressing (2) group (ANOVA with Dunnett’s post hoc test)

	Group	First visit		Intermediate visit			Last visit		
		Mean	StDev	Mean	StDev	p	Mean	StDev	p
Vis	1	0.98	0.05	0.96	0.09	NS	0.95	0.12	NS
	2	0.95	0.09	0.92	0.12	NS	0.96	0.09	NS
IOP (mmHg)	1	19.37	4.06	20.16	4.37	NS	19.00	4.69	NS
	2	21.00	4.93	17.23	4.73	NS	18.62	5.68	NS
MD (dB)	1	1.24	2.25	1.24	1.98	NS	1.97	2.71	NS
	2	2.03	1.95	5.78	3.40	0.002	5.97	3.54	0.001
sLV (dB)	1	2.86	1.24	2.78	0.91	NS	2.83	1.17	NS
	2	3.95	1.95	5.69	2.53	0.01	5.78	2.79	0.007
P50 (μV)	1	4.52	1.27	4.31	1.14	NS	4.24	1.09	NS
	2	4.22	1.09	4.01	1.01	NS	3.88	0.72	NS
N95 (μV)	1	6.03	1.39	5.98	1.59	NS	6.20	1.60	NS
	2	5.40	1.29	4.77	1.25	NS	4.62	0.93	0.02
PhNR (μV)	1	14.42	7.30	14.61	6.95	NS	14.55	7.42	NS
	2	14.92	5.39	13.30	6.01	NS	12.03	5.57	NS
PhNR ratio	1	0.248	0.026	0.255	0.028	NS	0.236	0.022	NS
	2	0.266	0.032	0.229	0.037	NS	0.217	0.032	NS
pRNFL (μm)	1	82.26	9.12	78.79	12.5	NS	78.21	10.27	0.03
	2	75.77	11.46	66.77	13.68	0.003	61.31	15.50	< 0.001
NFL (μm)	1	36.95	7.18	36.47	7.13	NS	37.79	7.09	NS
	2	31.92	6.86	26.46	9.63	< 0.001	27.15	8.36	0.002
mGCIPL (μm)	1	63.42	4.69	63.21	4.84	NS	61.79	4.54	< 0.001
	2	61.85	5.10	58.69	6.52	< 0.001	55.62	7.33	< 0.001
GCC (μm)	1	100.26	10.64	99.68	10.92	NS	99.47	10.58	NS
	2	93.46	11.27	85.46	15.31	< 0.001	82.85	14.50	< 0.001

**Fig. 2:**
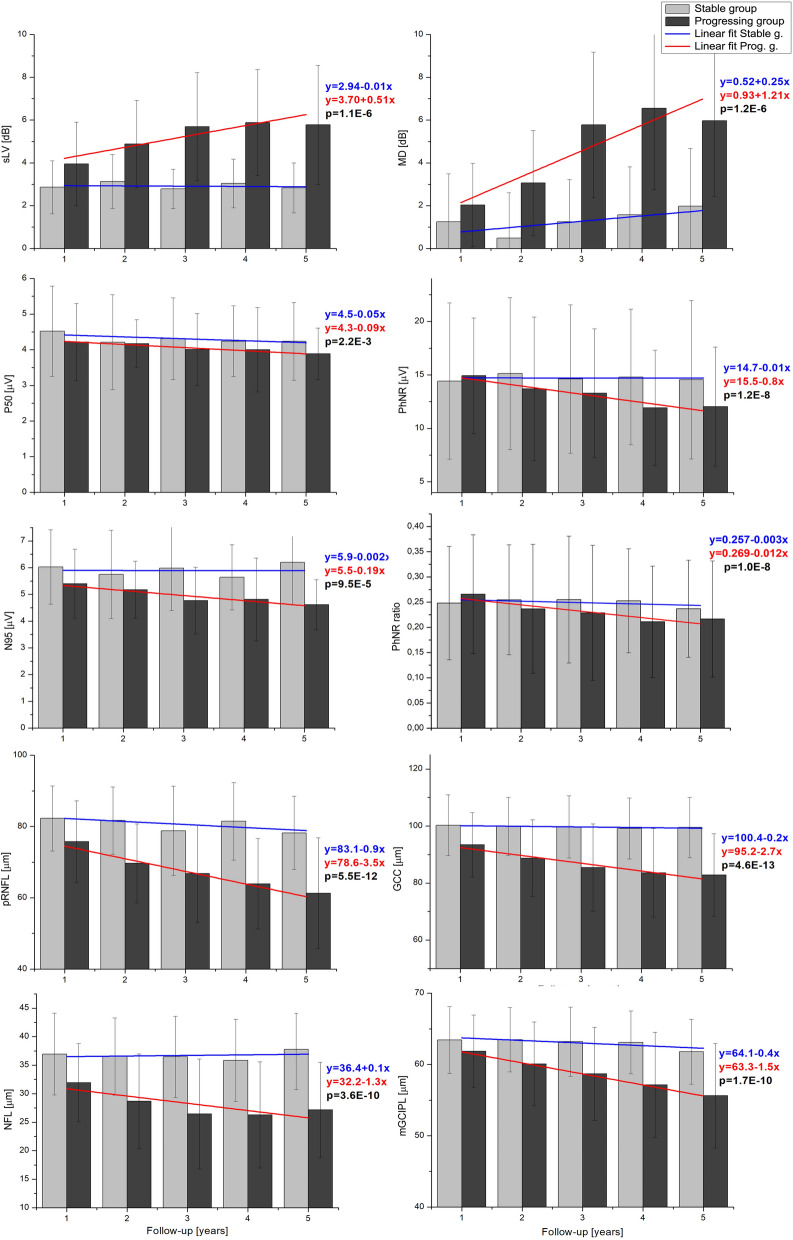
Mean value (± standard deviation) of the two SAP measures—mean defect (MD) and square root of loss variance (sLV), ERG measures—P50 amplitude (P50), N95 amplitude (N95), PhNR amplitude (PhNR) and PhNR amplitude ratio (PhNR ratio), and OCT measures—pRNFL thickness (pRNFL), macular NFL thickness (NFL), GCC thickness (GCC) and mGCIPL thickness (mGCIPL) at the follow-up visits for the stable and progressing group. Blue and red linear regression lines (*y* = *a* + *bx*) indicate the trend of changes over time for stable and progressive groups, respectively

The trend of change over time was further analysed and compared between the groups by applying linear regression curve to the mean values of each parameter at all the visits, as shown in Fig. 2. In the stable group, visual field indices, electrophysiological measures and OCT parameters showed more or less a horizontal fitted curve. In the progressing group, there was a steeper slope of the fitted line, indicating worsening of the values seen for the visual field indices, N95, PhNR, PhNR ratio and all the OCT parameters. The difference in slopes over time was significant for all the parameters except visual acuity and was highly significant for the GCC, pRNFL and mGCIPL thicknesses.

At the first visit, there were significant moderate correlations of PERG N95 and OCT measures with the visual field indices, with the highest negative correlation between pRNFL thicknesses and MD, and sLV (*r* = -0.50, *p* = 0.004 and r = -0−55, *P* = 0.001, respectively) (Table 3). At the last visit, the strength of association with visual field indices increased, more so for the OCT parameters (r>0.7, *p* < 0.001) than for the N95. The PhNR amplitude demonstrated significant, but modest correlation with the MD (*r* = -0.35, *p* = 0.047) (Table 3).

**Table 3:** Correlation of ERG and OCT measures with the visual field indices

		First visit		Last visit	
		MD (dB)	sLV (dB)	MD (dB)	sLV (dB)
P50 (μV)	Pearson Corr	−0.39	−0.30	−0.27	−0.14
	p	NS	NS	NS	NS
N95 (μV)	Pearson Corr	−0.44	−0.32	−0.57	−0.44
	p	0.012	NS	6.14E-04	0.012
PhNR (μV)	Pearson Corr	−0.15	−0.08	−0.35	−0.10
	p	NS	NS	0.046	NS
PhNR ratio	Pearson Corr	−0.13	0.04	−0.37	−0.11
	p	NS	NS	0.039	NS
pRNFL (μm)	Pearson Corr	−0.50	−0.55	−0.70	−0.61
	p	0.004	0.001	8.76E-06	2.30E-04
NFL(μm)	Pearson Corr	−0.43	−0.51	−0.73	−0.68
	p	0.013	0.00276	2.65E-06	1.62E-05
mGCIPL(μm)	Pearson Corr	−0.14	−0.17	−0.49	−0.45
	p	NS	NS	0.005	0.009
GCC (μm)	Pearson Corr	−0.36	−0.42	−0.67	−0.63
	p	0.044	0.017	2.55E-05	1.09E-04

Inter-session repeatability of ERG and OCT for the stable group between the first and second visit is shown as LoAs (Table 4) and graphically in the *Supplementary file* (Fig. S). OCT had better inter-session repeatability with smaller % LoAs (range from 3.6% for mGCIPL to 17.4% for pRNFL thicknesses) than ERG (range from 35.9% for N95 to 59.9% for PhNR amplitude). In eyes without progression (stable group), the coefficient of variation was calculated for repeated measurements at first and follow-up visits to compare the rate of inter-session variability between the ERG and OCT measures (Table 4). ERG measures (N95 and PhNR) showed higher measurement variability in relation to the mean than OCT measures. Among OCT measurements, the coefficient of variation was lower for macular parameters than for pRNFL thicknesses.

**Table 4:** Inter-session repeatability between visit 1 (V1) and 2 (V2), assessed by limits of agreement (LoA) and coefficient of variation (CoV) between all 5 visits for the ERG and OCT measures in the non-progressing eyes (n = 19)

	Mean V1	SD V1	Mean V2	SD V2	Mean diff V1-V2	SD diff	95%CI LoA	% LoA*	CoV (%)	SD CoV
P50 (μV)	4.52	1.27	4.21	1.33	0.31	0.95	−1.56 to 2.18	42.8%	14.7	7.4
N95 (μV)	6.03	1.39	5.75	1.65	0.28	1.08	−1.83 to 2.39	35.9%	11.9	7.1
PhNR (μV)	14.42	7.30	15.13	7.11	−0.71	4.51	−9.56 to 8.14	59.9%	23.6	11.2
PhNR ratio	0.25	0.11	0.25	0.11	−0.01	0.08	−0.16 to 0.14	59.3%	21.5	11.5
pRNFL (μm)	82.26	9.12	81.67	9.47	1.44	7.31	−12.88 to 8.75	17,4%	5.6	2.9
NFL (μm)	36.95	7.18	36.53	6.78	0.42	2.17	−3.83 to 4.67	11.6%	4.8	2.8
mGCIPL (μm)	63.42	4.69	63.47	4.50	−0.05	1.18	−1.46 to 1.36	3.6%	1.6	0.6
GCC (μm)	100.26	10.65	99.84	10.18	0.42	2.87	−5.21 to 6.05	5.6%	1.7	0.8

^*^ % LoA = ([1.96 × (SD V1-V2)]/(mean all V1 and V2) × 100); V1 = visit 1 and V2 = visit 2

To determine if the analysed parameters could be sensitive in distinguishing between the stable and progressing eyes, ROC analysis was applied to the data at the first visit (Table 5). The GCC and pRNFL thicknesses significantly discriminated between the progressing and non-progressing eyes with the area under the ROC curve of 0.72 and 0.71, respectively. The GCC thickness cut-off values of 97.5 µm had a 63% sensitivity and 85% specificity, whereas pRNFL thickness of 78.5 µm had 74% sensitivity and 77% specificity.

**Table 5:** Areas under the ROC curves at baseline for detecting progression

	AUC	StErr	p
IOP	0.42	0.10	NS
Vis	0.61	0.10	NS
MD	0.36	0.10	NS
sLV	0.31	0.09	NS
P50	0.57	0.11	NS
N95	0.65	0.11	NS
PhNR	0.47	0.11	NS
PhNR ratio	0.45	0.11	NS
pRNFL	0.71	0.10	0.046
NFL	0.70	0.10	NS
mGCIPL	0.64	0.10	NS
GCC	0.72	0.10	0.037

## Discussion

The purpose of this study was to evaluate PERG and PhNR in monitoring subjects and compare the ERG and structural OCT measures between the progressing and non-progressing eyes over a 4-year follow-up. The stable group had significant thinning for the pRNFL and mGCIPL thicknesses at the last follow-up visit only, whereas the progressing group showed significant deterioration in the visual field indices and OCT parameters at the intermediate visit, and significant changes for all parameters, except in the PhNR at the last visit. In progressing eyes, all measures (PERG N95, PhNR, RNFL and macular thicknesses) showed significantly steeper linear regression slopes, which were highly significant for the pRNFL and GCC thicknesses. At baseline, both OCT measures and N95 moderately correlated with the visual field indices, whereas at the last visit strong correlation with the visual field indices was present only for the OCT measures. The ROC curves showed that at the first visit the pRNFL and GCC thicknesses were the only measures that discriminated between the progressing and stable eyes.

The majority of previous reports address PERG and PhNR as an objective method to help in the early diagnosis of glaucoma or investigate the correlation of ERG measures with visual field parameters and structural changes [3–7, 9, 10, 18, 19]. Many glaucoma studies used a steady-state PERG, recorded with a faster reversal rate of pattern stimulus (typically 16 reversals per second (rps)) which generates a steady-state, sinusoidal waveform, whose period corresponds to the reversal frequency. The steady-state PERG reflects mainly spike-related ON pathway activity, whereas transient PERG (used in our study) receives nearly equal amplitude contributions from ON and OFF pathways with N95 reflecting spiking activity of ganglion cells and P50 non-spiking activity as well [20]. PERG and PhNR are measures of RGC integrity, and lowering of IOP in OHT and early glaucoma eyes was associated with an increase in PERG and PhNR amplitudes indicating an improvement in the inner retinal function [21–24]. It would be expected that there is weak to modest correlation of electrophysiological with structural measures and visual field as these assess different aspects of pathological process that do not occur at the same time (i.e. retinal dysfunction preceding cell death) [18, 25]. In addition, PERG and PhNR reflect function of the inner retina (PERG is a central response, while PhNR is a diffuse response from the whole retina, and therefore the two tests may offer different levels of information) [26], whereas SAP represents not only the retinal activity but also the activity of the whole visual pathway. OCT measures structural changes of the optic nerve, RNFL and macular parameters which can help clinician to distinguish the anatomic changes in glaucoma patients when compared with normal subjects [27]. Like ERG, the diagnostic ability of OCT is modest in suspect glaucoma and improves with the severity of glaucoma [28, 29].

Cross-sectional studies reported variable, usually weak to moderate correlation between PERG/PhNR and SAP/structural parameters [30–34] or even lack of correlation [25, 35]. Different stages of disease (e.g. OHT, suspect, early or advanced glaucoma) and a high variability of PERG/PhNR amplitudes in the normal population may account for different findings. This variability in normal subjects can affect the results that patients with glaucomatous visual field defect can still have normal PERG [25]. Similarly, we found a considerable overlap of PERG and PhNR amplitudes among OHT, suspect and early glaucoma patients at baseline (*Supplementary file*) and consequently absence of correlation or moderate correlation of the PhNR and N95 with the visual field parameters.

Several prospective studies assessed the ability of electrophysiological measures to predict progression in OHT or suspect glaucoma eyes using mainly steady-state PERG [36–39]. Bach et al. [40] comparing two steady-state PERG protocols with the same reversal rate (15 rps) showed that the PERGLA protocol using skin electrodes detected glaucoma similarly to the PERG ratio protocol (PERG to two check sizes of 0.8° and 16°) using corneal electrodes. The largest study including 120 eyes of 64 patients with OHT and the mean follow-up of 10.3 years found that 10% of eyes converted to glaucoma with visual field defect [39]. A PERG amplitude ratio (for standard/large checks reversing at 15 rps) had a significantly steeper mean negative slope over time in converters when compared to non-converters and detected glaucoma patients 4 years before visual field changes occurred. The PERG ratio showed an area under ROC curve of 0.75 (sensitivity of 75%, specificity of 76%) [39]. Ventura et al. [41] monitored RGC function in suspect glaucoma patients (with normal visual field) using PERGs to check alternating at 15 rps (PERGLA paradigm) over a mean of 5.7 years. The PERG amplitude showed a significant negative slope in 15% to 20% of suspect glaucoma eyes, while significant progression of SAP-MD was found in only 0% to 2% of eyes. Banitt et al. [42] evaluated longitudinal rates of change for the pRNFL thickness using OCT and PERG amplitude in suspect glaucoma patients. They found that patients with significantly reduced baseline PERG amplitude (≤ 50% of its age-adjusted normative value) had lower baseline RNFL thicknesses, and the fastest rate of RNFL thinning over the subsequent 5 years. In our study, at baseline visit, only the pRNFL and GCC thicknesses significantly discriminated between the stable and progressing eyes. Similarly, Siesky et al. reported that thinner mean RNFL thickness at baseline was associated with shorter time to visual field progression over the 5-year follow-up [43]. In a recent retrospective study including 357 glaucoma suspects with a follow-up of up to 5.7 years, faster thinning of pRNFL (-1.13 ± 0.85 µm/year) and mGCIPL (-0.71 ± 0.57 µm/year) thicknesses predicted development of visual field defects [44]. In the same study, the rate of change in average pRNFL (-0.27 ± 0.64 µm/year) and GCIPL (-0.19 ± 0.32 µm/year) thicknesses was significant over time also in glaucoma suspect eyes that did not show visual field changes [44]. In our study, a significant decline over time was found for pRNFL and mGCIPL thicknesses in the stable group as well. Longitudinal and cross-sectional analyses showed a consistent rate of approximately 0.2% per year of age-related thinning in NFL and GCC thicknesses [45], but presently commercially available OCT algorithms for monitoring progression do not incorporate thinning due to ageing effect.

The main goal of our study was to investigate the role of both ERG and OCT in monitoring glaucoma patients. In this study, the mean PhNR and N95 amplitude showed a significant negative slope for progressing eyes, but the OCT measures demonstrated even steeper linear slopes between the two groups and appear to be more useful in detecting progression. In addition, we recorded high variability of ERG measurements in stable eyes over time. The coefficients of variation for the non-progressing group were similar to those found by others [40, 46, 47]. These normalized coefficients of variation (11.9% for N95 and 23.6% for PhNR) were high compared with those for anatomical measures (1.6%-5.6%), limiting their sensitivity for detecting changes. However, a certain percentage of variability found for the pRNFL and mGCIPL thicknesses in the stable group may have been caused by true progression due to ageing effect, as both OCT measures showed a significant thinning at the last follow-up visit. Furthermore, the inter-session variability/repeatability was also calculated as LoAs for the first two visits in the stable group and showed that OCT had lower inter-session variability than ERG measurements. OCT mGCIPL thickness had the smallest test–retest variation, within ± 3.6% of the mean; the inter-session variation of PERG N95 amplitude was within ± 35.9% of the mean and of PhNR within 59.9% of the mean. Two studies reported much larger variation for PhNR of ± 88.4% and ± 148.3% of mean amplitude [17, 48].

One of the limitations of our study was the small number of mixed cases including subjects with OHT, suspect and early glaucoma patients compared to prospective studies including suspect glaucoma only. Furthermore, medical treatment has been changed in some patients to achieve lower intraocular pressure which may affect retinal function with a potential improvement in ERG responses [22]. The strength of our study was that clinical criteria for progression were used: change in the optic disc and/or visual field deterioration confirmed by trend analysis.

Although a considerable body of evidence exists that supports the usefulness of PERG and PhNR in predicting and detecting early glaucomatous damage, the present study shows that both have limited applicability in monitoring glaucoma progression, mainly due to high inter-session variability, which hinders detection of true changes over time from noise. Conversely, OCT measures show low inter-session variability and might have a better predicting value for early differentiation of progressing cases in clinical practice.

## Supplementary Information

Below is the link to the electronic supplementary material.

Supplementary file1 (DOCX 166 kb)

## Data Availability

Data are available from the corresponding author on reasonable request.
