# Supplementary material for: Monitoring for glaucoma progression with SAP, electroretinography (PERG and PhNR) and OCT
Source: Doc Ophthalmol. 2021 Oct 15;144(1):17–30. doi: 10.1007/s10633-021-09854-8 (PMC8882567; doi:10.1007/s10633-021-09854-8)
Supplement: Supplementary file 1 — Supplementary file1 (DOCX 166 kb) [file 10633_2021_9854_MOESM1_ESM.docx]

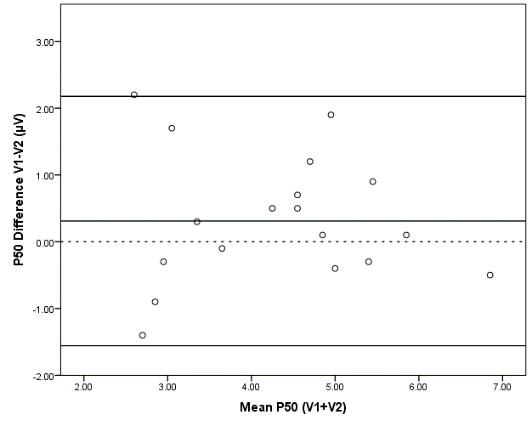


Mean – 1.96SD

Mean

Mean + 1.96SD


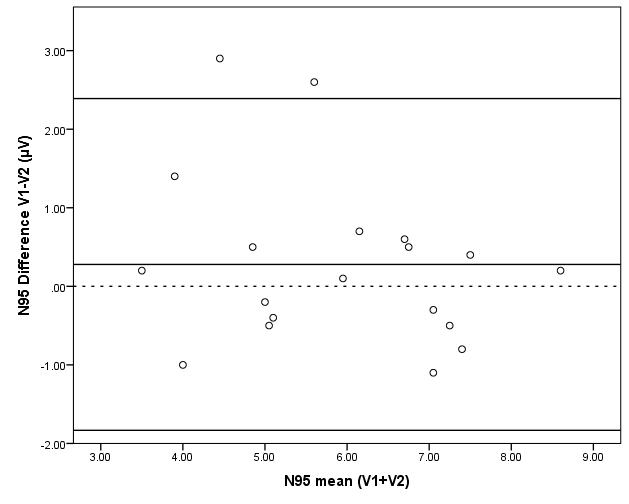


Mean – 1.96SD

Mean

Mean + 1.96SD


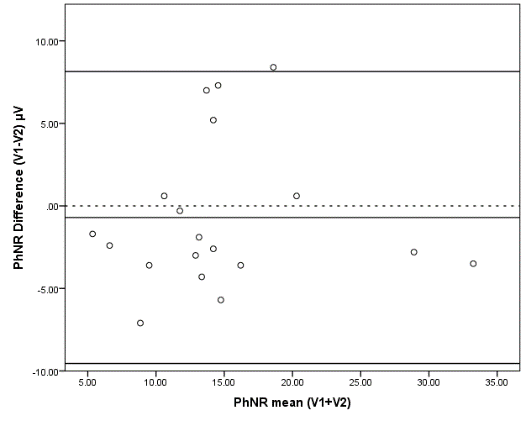


Mean – 1.96SD

Mean

Mean + 1.96SD


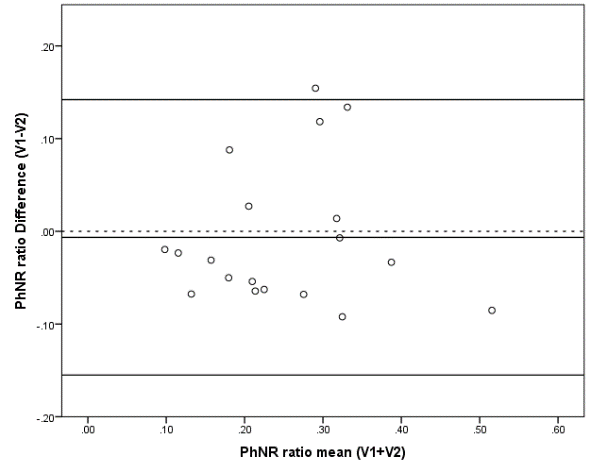


Mean – 1.96SD

Mean

Mean + 1.96SD


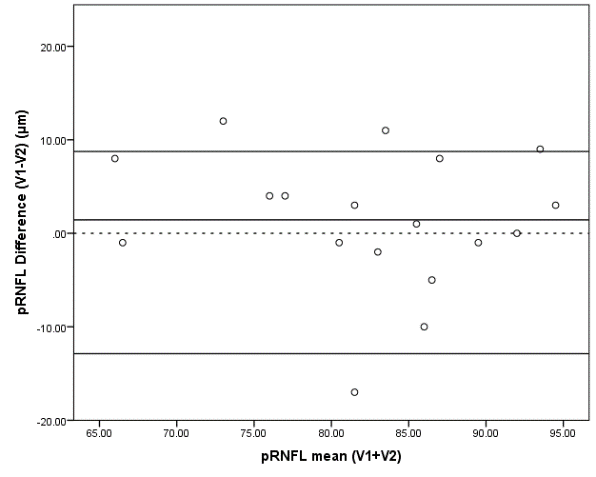


Mean + 1.96SD

Mean – 1.96SD

Mean


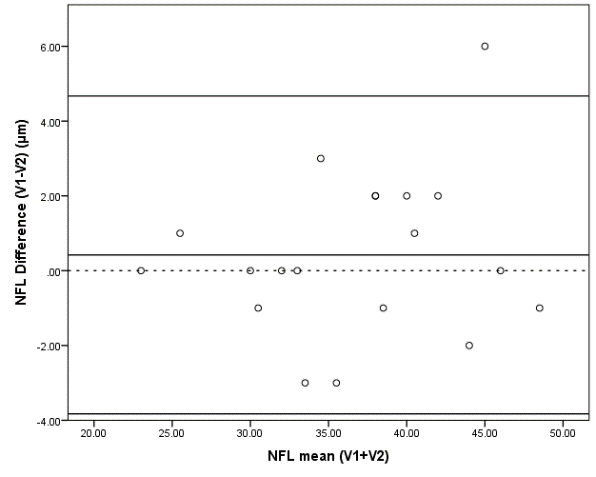


Mean – 1.96SD

Mean

Mean + 1.96SD


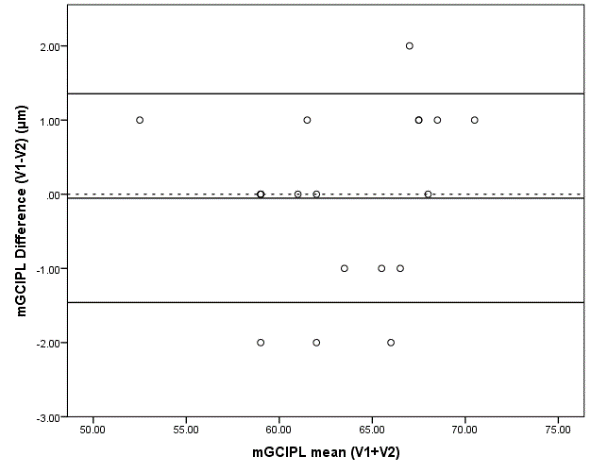


Mean – 1.96SD

Mean

Mean + 1.96SD


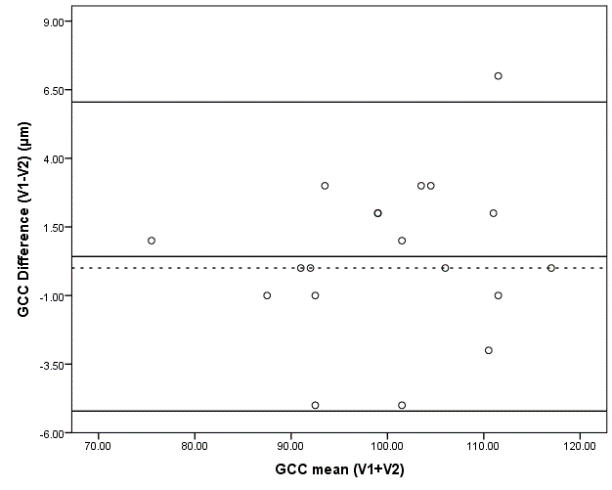


Mean – 1.96SD

Mean

Mean + 1.96SD

**Figure S:** Bland-Altman plots showing the inter-visit repeatability with the mean difference and 95% CI for the limits of agreement for the ERG and OCT measures in the non-progressing eyes for the first (V1) and second visit (V2)


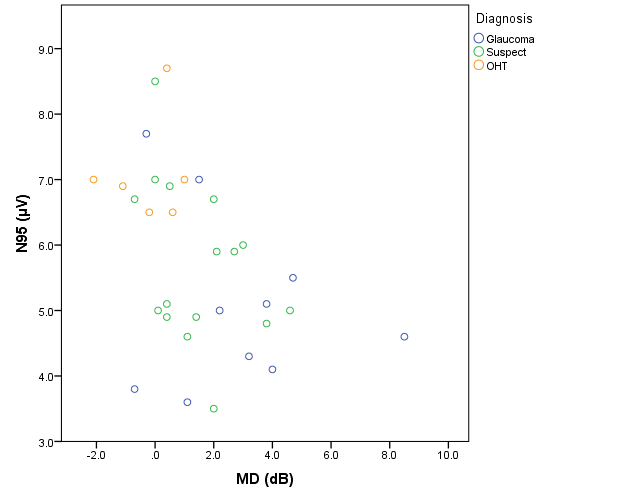


**Fig. S1** N95 amplitude and visual field mean defect (MD) in subjects with ocular hypertension (OHT) and patients with suspected and early glaucoma at baseline


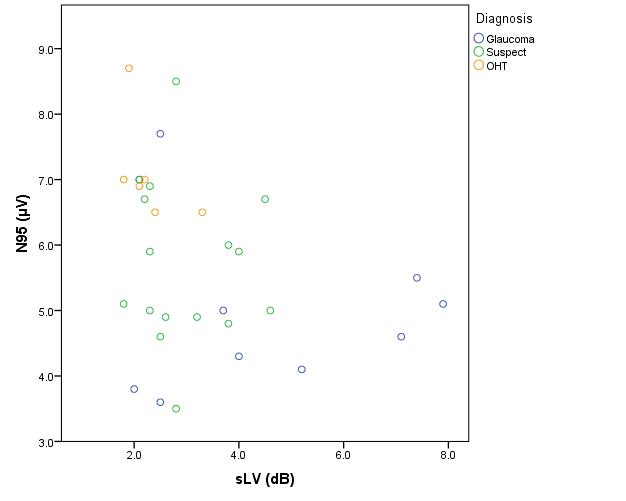


**Fig. S2** N95 amplitude and visual field square root of loss variance (sLV) in subjects with ocular hypertension (OHT) and patients with suspected and early glaucoma at baseline


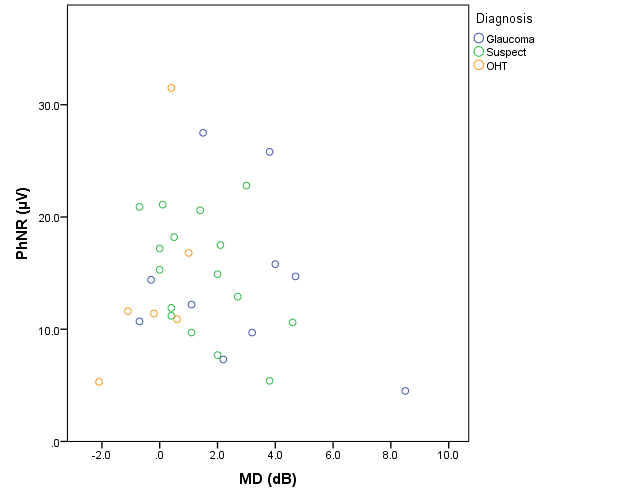


**Fig. S3** PhNR amplitude and visual field mean defect (MD) in subjects with ocular hypertension (OHT) and patients with suspected and early glaucoma at baseline


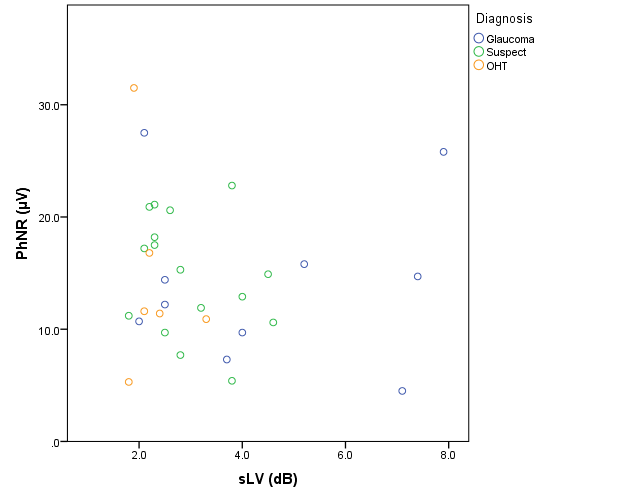


**Fig. S4** PhNR amplitude and visual field square root of loss variance (sLV) in subjects with ocular hypertension (OHT) and patients with suspected and early glaucoma at baseline
